# Isothermal Crystallization Kinetics of Poly(4-hydroxybutyrate) Biopolymer

**DOI:** 10.3390/ma12152488

**Published:** 2019-08-06

**Authors:** Ina Keridou, Luis J. del Valle, Lutz Funk, Pau Turon, Ibraheem Yousef, Lourdes Franco, Jordi Puiggalí

**Affiliations:** 1 Departament d’Enginyeria Química, Universitat Politècnica de Catalunya, Escola d’Enginyeria de Barcelona Est-EEBE, c/Eduard Maristany 10-14, 08019 Barcelona, Spain; 2 Center for Research in Nano-Engineering, Universitat Politècnica de Catalunya, Campus Sud, Edifici C’, c/Pasqual i Vila s/n, E-08028 Barcelona, Spain; 3 B. Braun Surgical, S.A. Carretera de Terrassa 121, 08191 Rubí (Barcelona), Spain; 4 ALBA Synchrotron Light Facility, Carrer de la Llum, 2-26, Cerdanyola del Vallès, 08290 Barcelona, Spain

**Keywords:** Poly(4-hydroxybutyrate), biodegradable polyesters, isothermal crystallization kinetics, secondary nucleation, spherulitic morphology, infrared microspectroscopy, synchrotron radiation

## Abstract

Thermal properties and crystallization kinetics of poly(4-hydroxybutyrate) (P4HB) have been studied. The polymer shows the typical complex melting behavior associated to different lamellar populations. Annealing processes had great repercussions on properties and the morphology of constitutive lamellae as verified by X-ray scattering data. Kinetics of isothermal crystallization was evaluated by both polarizing optical microscopy (POM) and calorimetric (DSC) measurements, which indicated a single crystallization regime. P4HB rendered banded spherulites with a negative birefringence when crystallized from the melt. Infrared microspectroscopy was applied to determine differences on the molecular orientation inside a specific ring according to the spherulite sectorization or between different rings along a determined spherulitic radius. Primary nucleation was increased during crystallization and when temperature decreased. Similar crystallization parameters were deduced from DSC and POM analyses (e.g., secondary nucleation parameters of 1.69 × 10^5^ K^2^ and 1.58 × 10^5^ K^2^, respectively). The effect of a sporadic nucleation was therefore minimized in the experimental crystallization temperature range and a good proportionality between overall crystallization rate (*k*) and crystal growth rate (*G*) was inferred. Similar bell-shaped curves were postulated to express the temperature dependence of both *k* and *G* rates, corresponding to the maximum of these curves close to a crystallization temperature of 14–15 °C.

## 1. Introduction

Poly(4-hydroxybutyrate) (P4HB) is a new generation biopolymer widely employed as a bioresorbable soft tissue reinforcement [1] due to its excellent mechanical and acceptable thermal properties, including high thermal stability [2]. Probably, the main drawback of P4HB is associated to its cost of production and purification, with chemical synthesis being discarded for large scale production [3]. P4HB is biosynthesized by a fermentation process (e.g., by means of recombinant *Escherichia coli* K12 [1,4] and used by microorganisms as energy reserve material.

The main characteristic of P4HB is its high elasticity which contrasts with the high stiffness of the most employed reabsorbable devices: polyglycolide (PGA) and polylactide (PLA). P4HB can be employed directly as a suture or knitted into a scaffold. The high elasticity confers upon P4HB special advantages for being employed as abdominal wall closure material. In addition, P4HB has interest in a wide range of biomedical applications, such as: patching materials for treatments of congenital cardiovascular defects, heart valves, vascular grafts and bulking agents [1]. In fact, the first commercial P4HB device was a bioresorbable suture launched in 2009 under the trademark of MonoMax^TM^ by B.Braun Surgical [5]. Subsequently, P4HB was commercialized by Tornier as a soft scaffold for tendon repair (BioFiber^®^) [6], it was also utilized in the production of P4HB meshes for hernia repair (Phasix^®^, [7]) and in reconstructive surgeries (GalaFLEX^®^, [8,9]).

P4HB sutures are processed through melt spinning in the form of both monofilament and multifilament fibers, although injection molding, extrusion and melt blowing have also been employed for other general applications. The material is characterized by a moderate degradation rate (i.e., intermediate between polyglycolide and polylactide), neutral and biocompatible degradation products, rapid tissue in-growth and low bacterial adherence. In vivo biocompatibility tests of P4HB are highly favorable since its hydrolysis yields 4-hydroxybutyric acid, which is a common metabolite in the human body [10]. Mechanical properties of P4HB are highly dependent not only on the degree of orientation [8], but also on the molecular weight [11]. Specifically, tensile strength, Young modulus and elongation at break can change from 50 MPa, 70 MPa and 1000% to 800 MPa, 670 MPa and 90%, respectively, Comparing to related aliphatic polyesters, like PGA (i.e., one methylene group unit) and PCL (i.e., five methylene group units) the mechanical properties of P4HB (three methylene group units) are clearly different [8,12,13]. Thus, a correlation between Young modulus and the elongation at break with the number of methylene groups cannot be found. Specifically, P4HB with an intermediate repeat unit length has the lowest modulus (i.e., typical values are 70 MPa, 6900 MPa and 400 MPa for P4HB, PGA and PCL, respectively) and the highest elongation (i.e., typical values are 1000%, <3% and 80% for P4HB, PGA and PCL, respectively) [1].

P4HB is a semicrystalline polymer with a structure defined by an orthorhombic unit cell (*a* = 0.775 nm, *b* = 0.477 nm, and *c* (fiber axis) = 1.199 nm) containing two antiparallel chain segments, a *P2_1_2_1_2_1_* space group and a slightly distorted all-trans conformation [14,15]. Molecular structure has been determined from X-ray diffraction patterns of annealed fibers [15,16] and from electron diffraction patterns of solution crystallized lamellae [15,16].

Enzymatic degradation studies have extensively been studied for poly(3-hydroxybutyric acid) and their copolymers with other poly(hydroxyalkanoate)s, including P4HB [17]. Results pointed out that degradation rate was highly influenced by the degree of crystallinity but also the crystal morphology (mainly the lamellar thickness) played a fundamental role. Therefore, control of crystalline dimensions during processing has a significant role for the subsequent biomedical applications of such materials.

Degradation studies performed with oriented and disoriented P4HB revealed also great differences that were a consequence of the polymer morphology and the orientation of the sample. Thus, disoriented fibers showed a highly significant loss of tensile strength after only four weeks of implantation (residual strength close to 18%) while oriented fibers can retain a good performance for a period of eight weeks, with the in vivo strength retention being close to 50% after 12 weeks [8]. Materials were degraded by surface erosion as revealed by inspecting the morphology and the scarce variation of the molecular weight of the remaining fiber fragments. Enzymatic degradation of lamellar crystals has also been evaluated [16] and we observed that lipase from *Pseudomonas sp.* and PHB depolymerase from *Pseudomonas stutzari* degraded single lamellae from the crystal edges rather than the chain-folded surfaces.

The studies performed with P4HB are mainly concerned with its applications, with those involved the physical characterization being clearly limited. This fact is not logical considering the above indicated peculiar mechanical behavior and the great variability of properties according to the processing conditions. The present work tries to delve into fundamental questions underling as the study of crystallization, from the melt and the amorphous states, and the determination of the corresponding kinetics. This aspect is crucial to be able to control the crystallinity of the material and its final properties during the processing. To the best of our knowledge and surprise, no concrete study covering this aspect has currently been performed with P4HB.

## 2. Materials and Methods

Commercially available sutures of P4HB (Monomax^TM^, USP 1) were kindly supplied by B. BRAUN Surgical S.A. Weight and number average molecular weights of Monomax^TM^ samples were 215.000 and 68.000 g/mol, as determined by size exclusion chromatography (GPC).

Molecular weight was estimated by size exclusion chromatography (GPC) using a liquid chromatograph (Shimadzu, model LC-8A Tokyo, Japan) equipped with an Empower computer program (Waters). A PL HFIP gel column (Polymer Lab, Agilent Technologies Deutschland GmbH, Böblingen, Germany) and a refractive index detector (Shimadzu RID-10A, Tokyo, Japan) were employed. The polymer was dissolved and eluted in 1,1,1,3,3,3-hexafluoroisopropanol (HFIP) containing CF_3_COONa (0.05 M) at a flow rate of 0.5 mL/min (injected volume 100 μL, sample concentration 2.0 mg/mL). Number and weight average molecular weights were calculated using polymethyl methacrylate standards.

Calorimetric data were obtained by differential scanning calorimetry with a TA Instruments Q100 series equipped with a refrigerated cooling system (RCS) operating at temperatures from −50 °C to 150 °C. Calibration was performed with indium. Experiments were conducted under a flow of dry nitrogen with a sample weight of approximately 5 mg. Basic thermal characterization was performed following a four run protocol consisting on a heating run (10 °C/min) a cooling run (10 °C/min) after keeping the sample in the melt state for one minute, a subsequent heating run (10 °C/min) of the melt crystallized sample and finally a heating run (10 °C/min) of a sample cooled at the maximum rate allowed by the equipment.

For the study of isothermal crystallization from the melt state the sample was heated up to 100 °C (i.e., around 30 °C above the melting peak) at a heating rate of 10 °C/min. The sample was held for 5 min at 100 °C to erase sample history and subsequently cooled at a rate of 50 °C/min to the selected isothermal temperature where it was kept until baseline was attained. For cold crystallization experiments samples were firstly quenched from the melt state and then heated to the selected isothermal temperature.

The spherulitic growth rate was determined by optical microscopy using a Zeiss Axioskop 40 Pol light polarizing microscope (Carl Zeiss, Göttingen, Germany) equipped with a Linkam temperature control system configured by a THMS 600 heating and freezing stage connected to a LNP 94 liquid nitrogen cooling system. Spherulites were grown from homogeneous thin films prepared by evaporation of dilute solutions of the polymer in HFIP (0.1 g/mL). The films were dried at vacuum until a constant weight was achieved. Small sections of these films were pressed or smeared between two cover slides to get thicknesses close to 10 μm. Subsequently, the samples were inserted into the hot stage and kept at 100 °C for 5 min to eliminate sample history effects. Crystallization from the melt was evaluated after a fast cooling to the selected crystallization temperature. The spherulitic growth rate was determined by optical microscopy taking images with a Zeiss AxioCam MRC5 digital camera (Carl Zeiss, Göttingen, Germany) every five min. The plot of the measured spherulite radius versus time allows deducing the growth rate at each selected temperature from the corresponding slope. A first-order red tint plate was employed to determine the sign of spherulitic birefringence under crossed polarizers.

Wide angle X-ray diffraction (WAXD) and Small angle X-ray scattering (SAXS) data were obtained at the NCD beamline (BL11) of the ALBA synchrotron facility (Cerdanyola del Vallès, Barcelona, Spain), by using a wavelength of 0.100 nm. A WAXD LX255-HS detector from Rayonix and an ImXPAD S1400 photon counting detector were employed. Polymer samples were confined between Kapton films. WAXD and SAXS diffraction patterns were calibrated with Cr_2_O_3_ and silver behenate (AgBh), respectively. The correlation function and the corresponding parameters were calculated with the CORFUNC program for Fibre Diffraction/Non-Crystalline Diffraction provided by the Collaborative Computational Project 13. Deconvolution of WAXD peaks was performed using the PeakFit 4.0 program.

Synchrotron-based infrared microspectroscopy measurements (transmission mode) have been performed at the infrared beamline MIRAS of ALBA synchrotron using the Hyperion 3000 microscope coupled to Vertex 70 spectrometer (Bruker, Germany) at 4 cm^−1^ resolution with 256 co-added scans per spectrum. In this case, the spherulites were grown from films prepared directly over infrared transparent windows of CaF_2_ of 13mm diameter and 0.5 thickness. All spectra were obtained using a single masking aperture size of 8 μm × 8 μm. In addition to the intrinsic quasi linear light polarization of synchrotron radiation, the synchrotron light was also polarized at 0° or 90° by a ZnSe holographic wire grid polarizer (Acal BFi Germany GmbH).

## 3. Results and Discussion 

### 3.1. Thermal properties of P4HB

**Figure 1:**
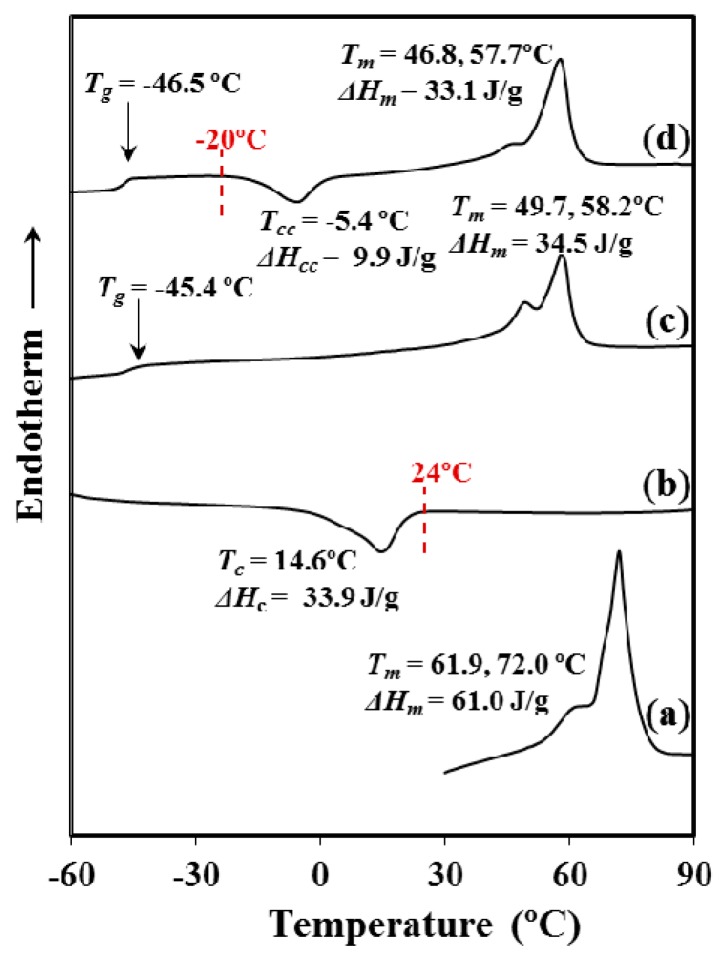
Sequence of calorimetric and heating runs performed with the initial commercial suture of poly(4-hydroxybutyrate (P4HB): (**a**) First heating run performed at 10 °C/min; (**b**) Cooling run at 10 °C/min after keeping the sample in the melt state for one min; (**c**) Heating run at 10 °C/min of the above melt crystallized samples and (**d**) Heating run at 10 °C/min of a sample quenched from the melt at the maximum rate allowed by the equipment.

Thermal behaviour of commercial P4HB sutures is shown in Figure 1, the following points are noticeable:

1. P4HB shows a complex fusion that depends on the processing conditions. Specifically, fusion of the processed suture is highly different from that determined from a melt crystallized (i.e., second heating run) and even a cold crystallized (i.e., third heating run corresponding to a melt quenched sample) sample. In all cases, a double melting point can be observed, but a remarkable shift to higher temperatures is detected for the initial suture in comparison with the melted and cold crystallized samples. Note that the temperature of the maximum intensity peak increases from 58 °C to 72 °C and the temperature of the lower peak that moves from 47–50 °C to 62 °C. In summary, temperature increases by approximately 10 °C for both melting peaks. These peaks can be associated to a typical lamellar reordering process where thinner lamellae become thicker [18]. Note that P4HB does not exhibit polymorphism and consequently endothermic peaks associated to structural changes can be discarded.
2. Crystallization from both the melt and the glassy state (i.e., that attained by a fast cooling to temperatures lower than the glass transition temperature) is a complex process that requires long time and a thermal annealing process to improve crystallinity. Note that the crystallization enthalpy determined for a slow cooling run (10 °C/min, Figure 1) from the melt state was around 57% of the melting enthalpy determined for the commercial annealed sample (i.e., 34.5 J/g with respect to 61 J/g). Nevertheless, a significant crystallization takes place even if the sample is quenched from the melt (i.e., (33.1–9.9) J/g was determined in third heating run given in Figure 1, a value that corresponds to a 38% of the maximum enthalpy).
3. Significant differences are observed between second and third heating runs. Amorphous content is logically higher for the fast crystallized sample as evidenced from the increase of *C_p_* (i.e., 0.2718 J/g·°C with respect to 0.3233 J/g·°C) at the glass transition temperature (−46.5 °C). Calorimetric (DSC) traces indicate also a lower perfection of the thin lamellar crystals for the quenched sample since the lamellar reordering process was enhanced (i.e., a lower relative intensity for the low temperature melting peak is derived in this case).
4. Final crystallinities attained for samples crystallized from the melt and the glassy states become similar due to the cold crystallization process that only develops during heating in the second case.

The given results point out the great difficulty of P4HB to render a high crystallinity and also to develop perfect crystals. Probably, the high polymer molecular weight renders a high melt viscosity and a large number of chain entanglements. Logically, low chain mobility is expected and consequently, there is great difficulty to both complete crystallization and the development of thick crystals with low defects during the final reordering process.

**Figure 2:**
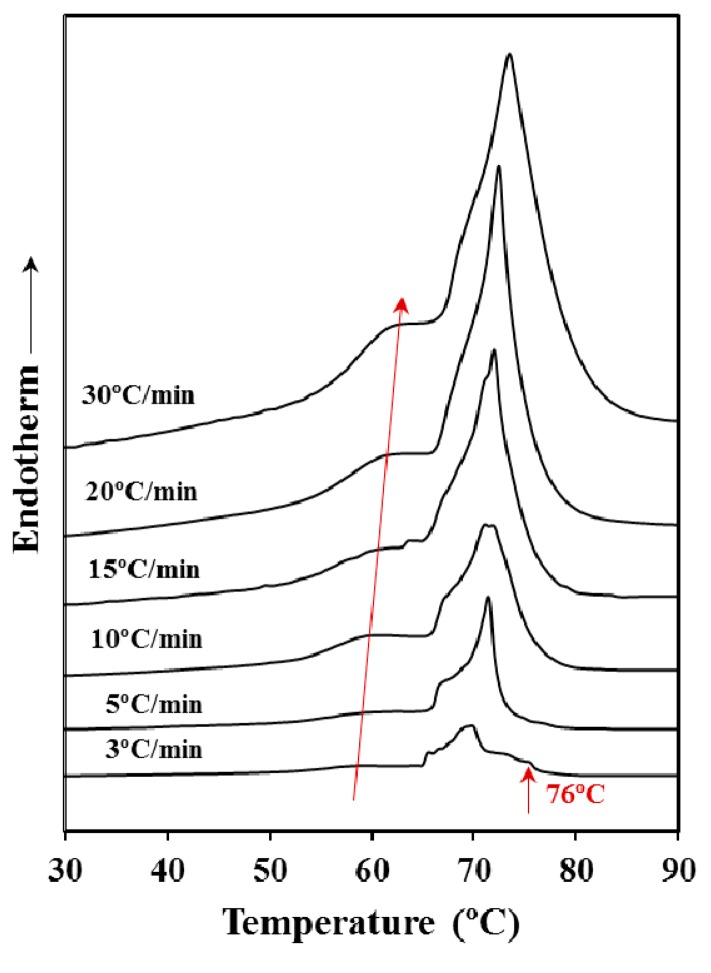
Calorimetric (DSC) heating runs performed at the indicated rates with the commercial P4HB (Poly(4-hydroxybutyrate)) suture.

Figure 2 shows that the heating rate has a clear influence on the melting process even if the sample was previously annealed. Note the great difference in the melting process between samples heated at rates of 3 and 30 °C/min and the gradual change observed for intermediate rates. Thus, the low temperature melting peak gradually moved to the right (i.e., to higher temperature) and increased its relative intensity with respect to the larger peak as the heating rate was increased. These features suggest a worse heat transmission (i.e., the increase of the peak temperature) and a lower capacity to suffer a reordering process during heating that lead to thicker lamellae with an increased melting point. More interestingly, the high temperature peak appears clearly split giving rise to multiple peaks when the heating rate was the lowest one (i.e., 3 °C/min). Specifically, it should be pointed out that a peak associated to highly reorganized lamellae appears clearly defined at a temperature close to 76 °C.

### 3.2. X-ray Diffraction Analysis of P4HB

**Figure 3:**
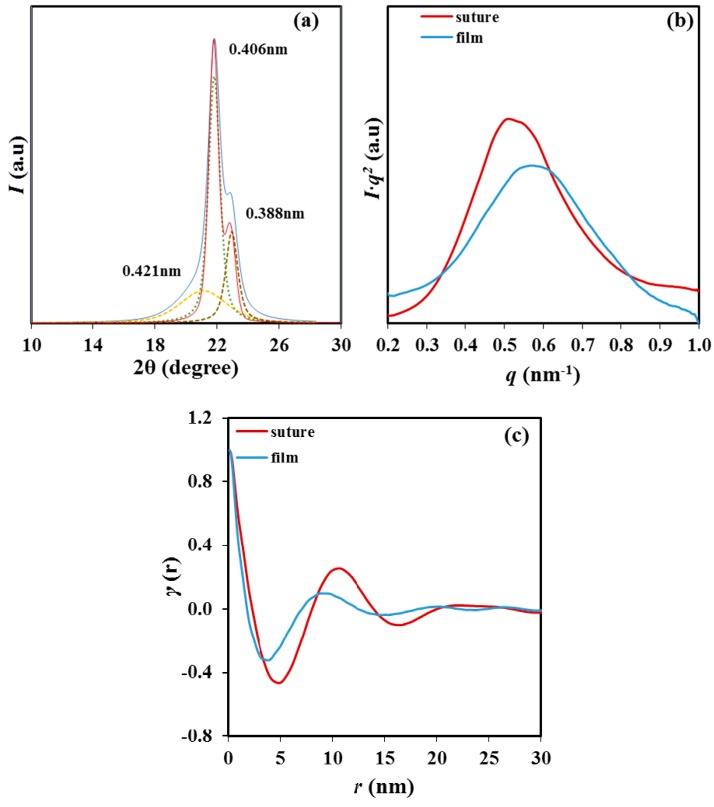
Wide angle X-ray diffraction (WAXD) profiles (**a**); Small angle X-ray scattering (SAXS) profiles (**b**) and correlation functions (**c**) of the initial commercial P4HB suture (red line) and a P4HB melt crystallized sample (blue line). Deconvoluted WAXD peaks are only indicated for the melt crystallized sample.

WAXD diffraction patterns of P4HB showed two predominant peaks at 0.406 and 0.388 nm (i.e., those corresponding to the (110) and (200) reflections of the reported orthorhombic structure) independently of the way as the polymer was prepared (Figure 3a). Nevertheless, some minor differences between the profiles of the original suture and the melt crystallized sample were found. These mainly corresponded to the broad amorphous halo centered at 0.421 nm that could only be clearly observed in the profile of the melt crystallized sample. On the contrary, large differences were found in the SAXS profiles (Figure 3b) where peak values of 9.5 nm to 10.5 nm were determined for the melt crystallized film and the processed suture. These values demonstrated the large differences on the supramolecular order (i.e., lamellar morphology) that could be induced by the thermal treatment. Diffraction peak of the melt crystallized film was less intense and broader indicating a worse contrast between the electronic densities corresponding to the amorphous lamellar surface and the crystalline lamellar core and even a large dispersity of the lamellar thickness [19].

Specific contributions of the crystalline lamellar core and the amorphous folding surfaces were determined through the *γ* (*r*) one—dimensional correlation function [20]:

(1) $$\gamma r=\;\int_{0}^{\infty }q^{2}I\left(q\right)\cos\left(qr\right)dq/\int_{0}^{\infty }q^{2}I\left(q\right)dq$$

where *I*(*q*) is the intensity at each value of the *q* scattering vector (=[4*π*/*λ*] sin *θ*, with *λ* and *θ* being the wavelength and the Bragg angle, respectively).

The correlation function (Figure 3c) showed better defined peaks for the processed suture as a consequence of the higher contrast between electronic densities of crystalline and amorphous phases. Calculated values of the long period, *L_γ_*, the amorphous layer thickness, *l_a_* and the crystalline lamellar thickness, *l_c_*, indicated an increase of all parameters after the annealing process. Specifically, changes from 8.8 nm to 10.5 nm, 1.7 nm to 2.6 nm and 7.1 nm to 7.9 nm were determined for *L_γ_*, *l_a_* and *l_c_*, respectively. Therefore, the crystalline region increased significantly and was in agreement with the increase of the melting temperature. At the same time, the dimension of the folding surface increased and became more disordered as deduced from its lower electron density (i.e., higher contrast). Changes are clarified by considering the crystallinity within the lamellar stacks (i.e., *X_c_*^SAXS^ = *l_c_*/*L_γ_*), which decreased from 80.6% to 75% after annealing as a consequence of the major impact caused by the disordered lamellar surface. 

### 3.3. Equilibrium Melting Point of P4HB

Equilibrium melting temperature (*T_m_*^0^) is a crucial parameter to perform an analysis of the crystallization process and specifically to evaluate the nucleation capacity of the surface of growing crystals as well as the degree of supercooling (*T_m_*^0^ − *T_c_*) at which crystallization is taking place. This temperature is associated to the theoretical fusion of crystals having an infinite thickness and can be estimated through the Hoffman-Weeks extrapolation [21]. This is a commonly accepted method due to its simplicity and straightforward experimental implementation. The method is based on Equation (2), which was deduced from a combination of the well-known Gibbs-Thomson equation and secondary nucleation theory [22]. The equation indicates that the melting temperature, *T_m_*, of a crystal varies with the temperature at which it was formed due to variation of the lamellar thickness. Specifically, the equation relates the melting and the crystallization temperature, *T_c_*, through the equilibrium melting temperature and the thickening coefficient, *γ*, defined as the ratio between the thickness of the grown crystal, *l_c_*, and the initial thickness of a “virgin lamella” l_g_*:

*T_m_* = *T_m_*^0^ (1 − 1/*γ*) + *T_c_*/*γ* (2)

**Figure 4:**
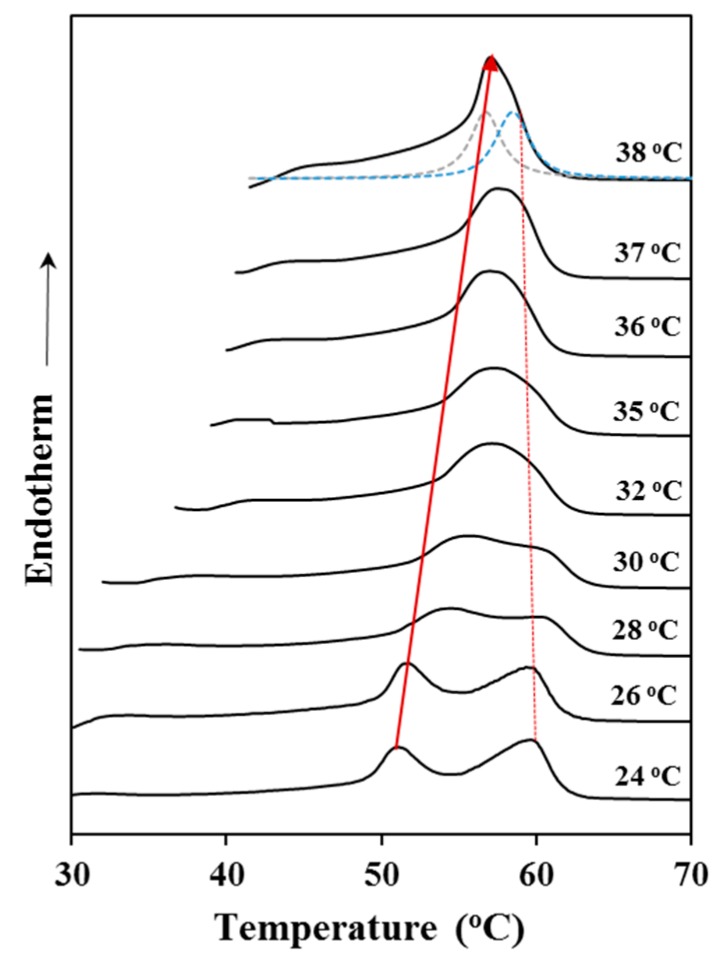
DSC heating runs of P4HB previously crystallized at the indicated temperatures.

A straight line can be obtained by plotting *T_m_* as a function of *T_c_*, with the equilibrium temperature corresponding to the intersection of this line with the *T_m_* = *T**_c_*** line. The validity of Equation (2) implies that lamellar crystals thicken at a specific crystallization temperature, which also influences the thickening parameter. Figure 4 shows the evolution of the melting peak of P4HB when it was crystallized at different temperatures. It is clear that the low temperature melting peak, which is associated to the initial melt of crystallized lamellae, shifted to higher temperatures with increasing crystallization temperature. On the contrary, the high temperature melting peak remains at a practically constant temperature since it corresponds to the fusion of reordered lamellae that are mainly formed during the heating process. Note also that at high crystallization temperatures the two melting peaks appear practically overlapped giving the false impression that the high temperature peak moves to lower temperatures. Deconvolution of DSC profiles (as shown for the crystallization performed at 38 °C) allowed determining the peak temperatures used for the Hoffman-Weeks plot. In addition, the relative intensity of the former peak increased as the crystallization temperature did as a consequence of the greater stability of the corresponding thin crystals (i.e., lower capacity to undertake a lamellar reordering process).

**Figure 5:**
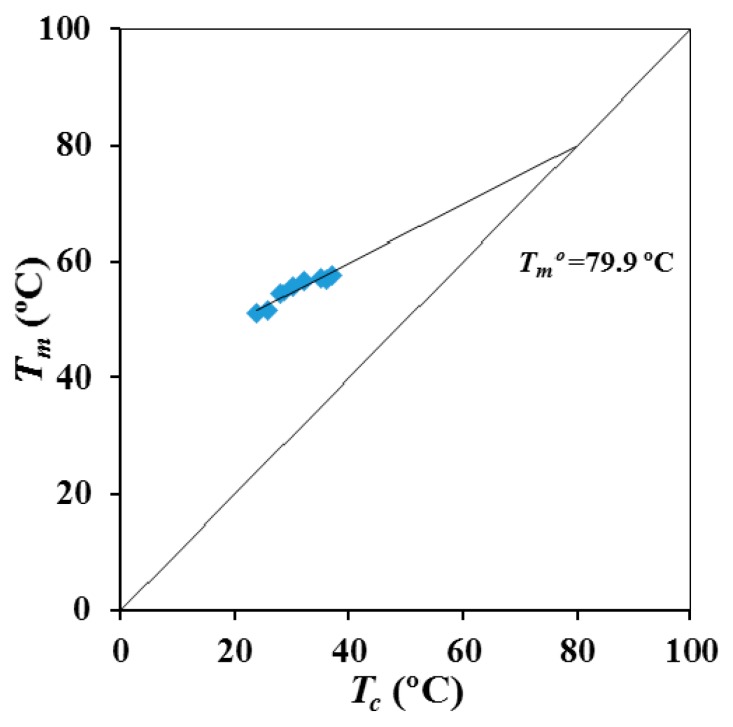
Hoffman-Weeks plot for P4HB considering the temperatures of its first melting peak.

Figure 5 shows the Hoffman-Weeks plot obtained from the temperature evolution of the former melting peak. Equilibrium melting temperature was 79.9 °C, a slightly higher value than the maximum peak temperature (76 °C) detected in the slow heating run of the commercial suture (Figure 2). 

### 3.4. Isothermal Crystallization Kinetics of P4HB Evaluated by Calorimetric Data

Kinetic crystallization analysis of P4HB was performed by cooling the samples from the melt state at selected isothermal temperatures and also by isothermal cold crystallization since in this case the polymer was not completely crystallized during the previous fast cooling from the melt. Note however, that cold crystallization should proceed under constrains imposed by the great crystalline fraction developed during the cooling run.

**Figure 6:**
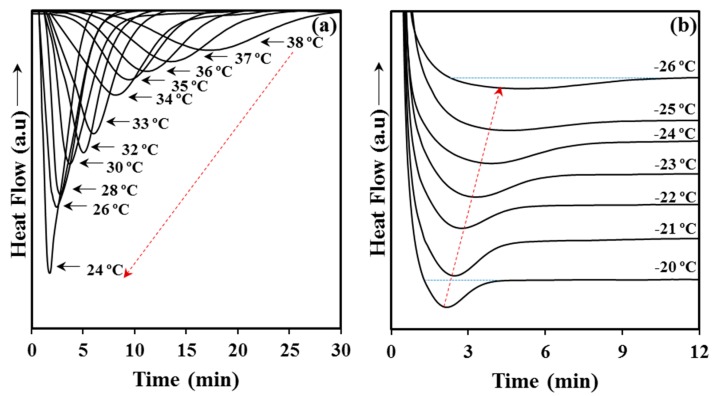
Exothermic DSC peaks corresponding to the isothermal crystallization from the melt state performed between 24 °C and 38 °C (**a**) and the cold crystallization performed between −26 °C and −20 °C (**b**).

Crystallization experiments from the melt state were successfully carried out in the narrow 24–38 °C temperature interval. Experimental problems concerning the time required to get a complete crystallization limited the highest temperature, while the lowest one was selected in order to avoid any trace of crystallization before reaching this temperature. The DSC cooling run shown in Figure 1 indicates the impossibility to start at temperatures lower than 24 °C. Figure 6a shows the variation of the DSC exothermic peaks with crystallization temperature. Logically, these peaks became narrower and shifted to lower times as the crystallization temperature decreased. Cold crystallization experiments were performed in the interval between −26 °C and −20 °C where peaks were still detectable (Figure 6b). Note that at lower temperatures the exothermic process required long times, where broad peaks were therefore derived, whereas at higher temperatures crystallization took place before reaching the selected temperature (see for example the temperature corresponding to the start of cold crystallization in Figure 1d). In general, cold crystallization exothermic peaks had low intensity as consequence of the previous crystallization during quenching and the final reduced amorphous fraction in the sample. Peaks broadened and shifted to higher times with the decrease of the selected crystallization temperature.

**Figure 7:**
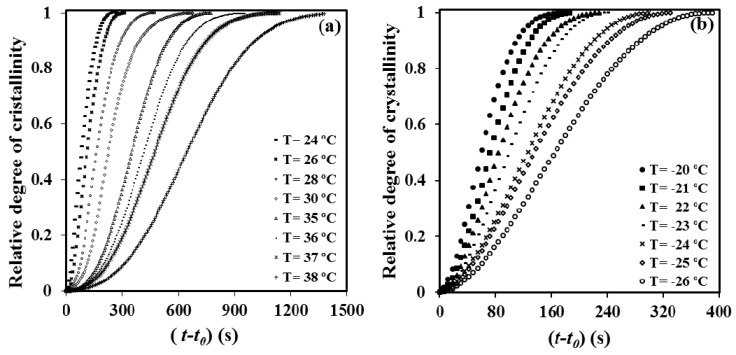
Evolution of the relative crystallinity over time for isothermal crystallizations of P4HB at the indicated temperatures. (**a**) Samples coming from the melt state; (**b**) Glassy sample obtained from a fast cooling from the melt.

The time evolution of the relative degree of crystallinity, *χ*(*t* − *t*_0_), was determined from the corresponding crystallization exotherms (Figure 7) through the ratio area: of the exotherm up to time *t* − *t*_0_ divided by the total exotherm area, i.e.,

(3) $$\chi \left(t-t_{0}\right)=\int_{t0}^{t}(dH/dt)dt/\int_{t0}^{\infty }(dH/dt)dt$$

where *dH*/*dt* is the heat flow rate and *t*_0_ the induction time. The development of crystallinity always showed a characteristic sigmoidal dependence on time, as plotted in Figure 7a,b for melt and cold crystallization experiments, respectively. These data were analyzed assuming the well-known Avrami equation [23,24] for primary crystallization:

1 − (*t* − *t*_0_) = exp[−*Z* (*t* − *t*_0_)*^n^*] (4)

where *Z* is the temperature-dependent rate constant and *n* the Avrami exponent whose value varies according to the crystallization mechanism. A normalized rate constant, k = *Z*^1/n^, is usually evaluated for comparison purposes since its dimension (time^−1^) is independent of the value of the Avrami exponent. 

**Figure 8:**
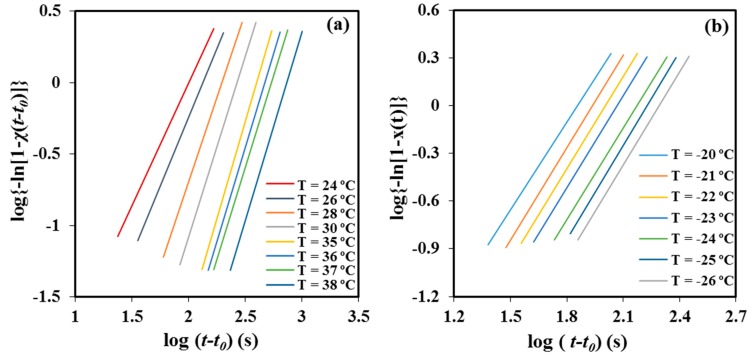
Avrami plots obtained from isothermal melt (**a**) and cold (**b**) crystallizations.

Table 1 summarizes the main kinetic parameters of the primary crystallization process from the melt state, as deduced from the plots of log{−ln[1 − *χ*(*t* − *t*_0_)]} against log(*t* − *t*_0_) (Figure 8a). The values of the Avrami exponent lay in a narrow range, from 2.35 to 2.62, with 2.56 being the average value. In general, the exponent increased with the crystallization temperature and suggests the occurrence at the higher temperatures of a predetermined (heterogeneous) nucleation with spherical growth under geometric constraints since the theoretical value should be equal to 3. Both sporadic (heterogeneous) and homogeneous nucleation can be clearly discarded as a higher exponent, close to 4, should be derived. Furthermore, homogeneous nucleation usually requires high undercooling, which is not the case.

Table 1 shows also that rather constant exponents between 1.84 and 1.97 (mean value of 1.93) were determined from the Avrami analysis of the cold crystallization process (Figure 8b). In this case, as above explained, crystallization must take place under high constrictions due to existence of a high ratio of P4HB crystallites produced during the previous cooling process. The value of the exponent became clearly lower than that determined from melt crystallizations and its temperature dependence did not follow any defined trend. 

**Figure 9:**
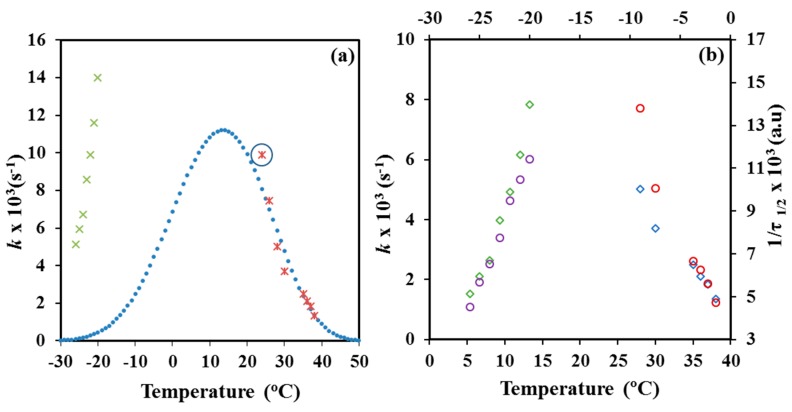
(**a**) Experimental (**×**) and simulated (**●**) temperature dependence of the overall crystallization rate of P4HB for isothermal melt crystallization. For the sake of completeness experimental cold crystallization data are also plotted (×); (**b**) Temperature dependence of the overall crystallization rates (**◊**, ◊) and the reciprocal crystallization half-times (о, о) of P4HB for melt and cold crystallizations.

*Z* and *k* values evaluated from both kinds of crystallizations are also summarized in Table 1. Logically, the crystallization rate took the lowest values at the highest and the lowest assayed temperatures as consequence of the restricted effective nucleation and the limited chain mobility, respectively. Maximum rate is expected between −20 °C and 24 °C considering a typical bell-shaped dependence with the temperature, as then will be explained. Figure 9a shows the variation of the overall crystallization rate with crystallization temperature. Melt crystallization data define the right side of the bell-shaped curve that is governed by the secondary nucleation process. Values of cold crystallization were not consistent with the typical representation since overall crystallization rates under the indicated constrictions where significantly higher than the expected for a normal growth.

The values of the corresponding reciprocal crystallization half-times (1/*τ*_1/2_), calculated as the inverse of the difference between crystallization start time (i.e., *t*_0_) and the time required to achieve a relative crystallinity of 0.5, are also given in Table 1. This parameter is a direct indicator of the crystallization process, and therefore can be used to check the accuracy of Avrami analysis by comparison with the theoretical kinetic value (i.e., 1/*τ*_1/2_ = (*Z*/ln2)^1/*n*^). Figure 9b demonstrated also the validity of the Avrami analysis for melt and cold crystallization since temperature evolution of the overall crystallization rate, *k*, was very similar to that found for the experimental crystallization data.

### 3.5. Spherulitic Morphologies of P4HB

**Figure 10:**
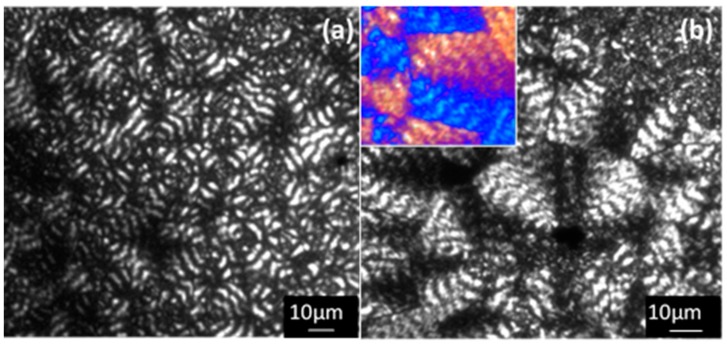
Optical micrographs showing P4HB spherulites crystallized at 36 °C (**a**) and 47 °C (**b**). The inset of (**b**) corresponds to a micrograph taken with a red tint plate.to determine the spherulite sign.

Crystallization of P4HB from the melt state rendered spherulitic morphologies with a banded texture (Figure 10a) at all assayed temperatures (i.e., between 36 °C and 49 °C). This kind of morphology is usually observed in the crystallization of different polymers and has merited different explanations based on: the development of interlamellar screw dislocations [25,26], the continuous twisting of lamellae [27], the rhythmic growth derived from the presence of depletion zones in the growth front [28] and the presence of different polymorphic structures [29]. It has also been postulated that final morphology is a consequence of a balance between the diffusion rate (*v_d_*) of the melted polymer and the crystallization rate (*v_c_*) [30,31]. These rates are defined by:

*v_d_* = d/dt[6*D*(*T_c_*)*t*]^1/2^ (5)

*v_c_* = *G*_0_ × exp[−U*/(*R* ×(*T_c_* − *T*_∞_))]× exp[*K_g_/T_c_*Δ*Tf*] (6)

where D is the whole chain diffusion constant, *T_c_* is the crystallization temperature, *t* is the whole diffusion time, *G*_0_ is a preexponential factor, generally assumed to be constant, U* is the transport activation energy, *T*_∞_ is the temperature at which all motions are associated with viscous flow cease, *f* is a factor which corrects for variation in the heat of fusion with temperature below the equilibrium melt point and taken as 2*T*/($T_{m}^{0}$ + *T*_∞_), Δ*T* is the supercooling degree, and *K_g_* is the secondary nucleation parameter which is related to the growth regime. Banded morphologies are usually observed when *v_d_* becomes lower than *v_c_* [30,31,32]. Note that independently of a possible lamellar twisting, a slow diffusion rate together with the volume shrinkage during solidification should cause a depletion zone at the growth fronts. Therefore, ridge and valley topographic textures with different width should be derived, it is also indicated in the crystallization of the related poly(3-hydroxybutyrate) [31] that banding with highly zigzag irregularities is formed at the highest crystallization temperature range. 

The observed spherulitic morphologies clearly revealed an increase of the zigzag irregularities of each band when temperature was increased, as well as, the differences between the width of birefringent and non-birefringent bands (i.e., the non-birefringent bands became narrower) (Figure 10). Spacing between bands was always close to 4–5 μm, a characteristic decrease with a temperature was not detected, probably as a consequence of the reduced temperature range at which spherulites were studied. Spherulites always showed a negative birefringence (inset of Figure 10b) characteristic of aliphatic polyesters.

### 3.6. Crystal Growth Rate and Primary Nucleation of P4HB

Kinetics of crystallization of P4HB from the melt state could be studied by optical microscopy but not for the glassy state since a high crystallinity was developed during the previous cooling run. The study of the development of spherulites was therefore precluded despite some cold crystallization taking place when samples were heated at the appropriate temperatures.

**Figure 11:**
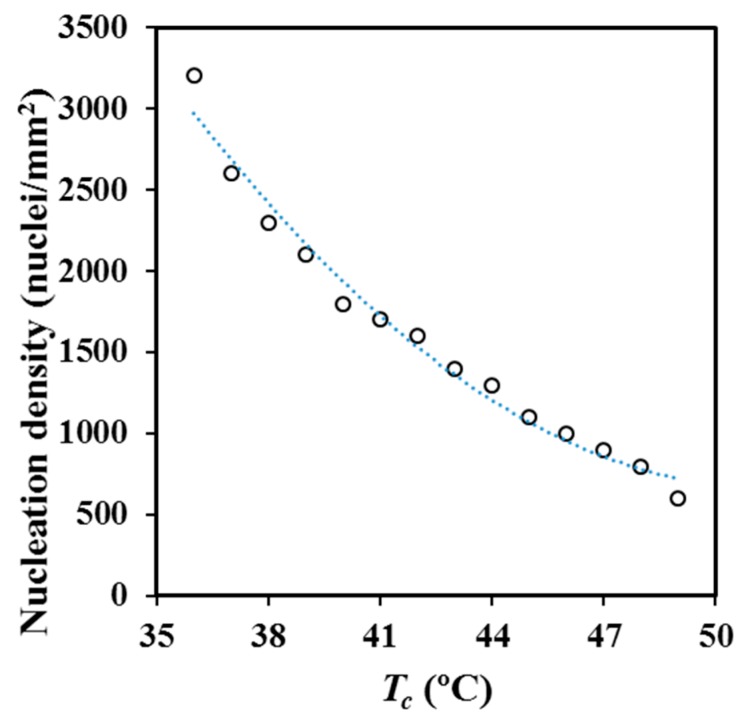
Temperature dependence of the primary nucleation density for crystallization performed from the melt state.

**Figure 12:**
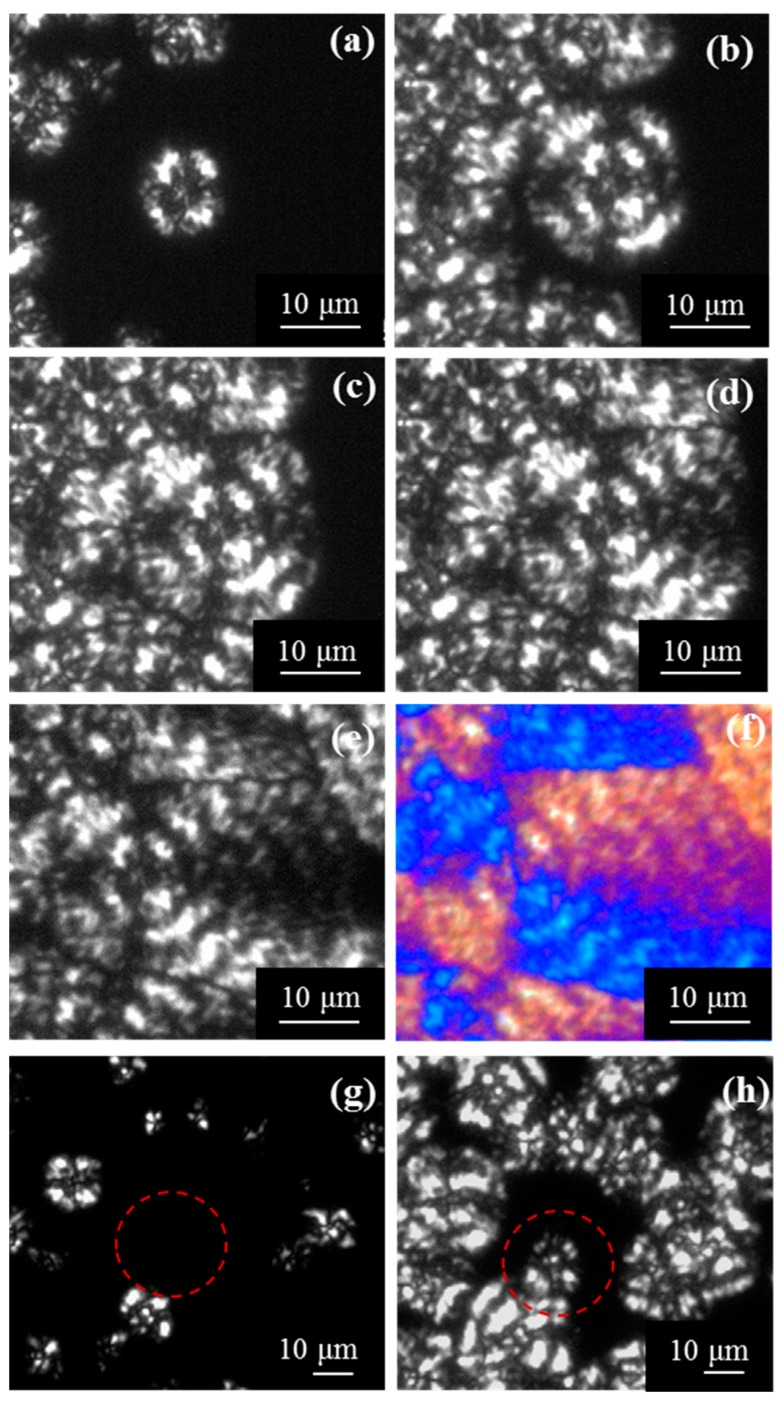
Optical micrographs showing P4HB spherulites crystallized at 47 °C for (**a**) 100 min, (**b**) 150 min, (**c**) 180 min, (**d**) 250 min, (**e**) 350 min and (**f**) 350 min. In the case of (**f**), the micrograph has been taken with a red tint plate. Comparison of micrographs taken after 47 min (**g**) and 80 min (**h**) for the crystallization performed at 46 °C. The dashed red circle points out the region where the apparition of a new spherulite is clear.

Figure 11 and optical micrographs given in Figure 12 show the great change of the nucleation density for melt crystallization when the selected isothermal temperature was varied, even in the very narrow temperature interval at which experiments could be performed (i.e., between 36 °C and 49 °C). Nucleation densities were determined by counting the number of spherulites observed in representative areas of optical micrographs. Primary nucleation density was exponentially increased from 600 nuclei/mm^2^ to 3200 nuclei/mm^2^ when crystallization temperature was reduced from 49 °C to 36 °C. Crystal grown measurements were not possible at lower temperatures than 36 °C due to the small size of the derived spherulites.

The number of spherulites increased during the isothermal crystallization process (as shown in Figure 12 where micrographs taken at different crystallization times for an isothermal process are compared). Therefore, P4HB follows a thermal crystallization (i.e., the number of active nuclei increased with time), probably as a consequence of an heterogeneous and sporadic nucleation since an alternative homogeneous nucleation can be discarded considering both the low degree of supercooling and the Avrami exponent values deduced from the calorimetric analysis. 

**Figure 13:**
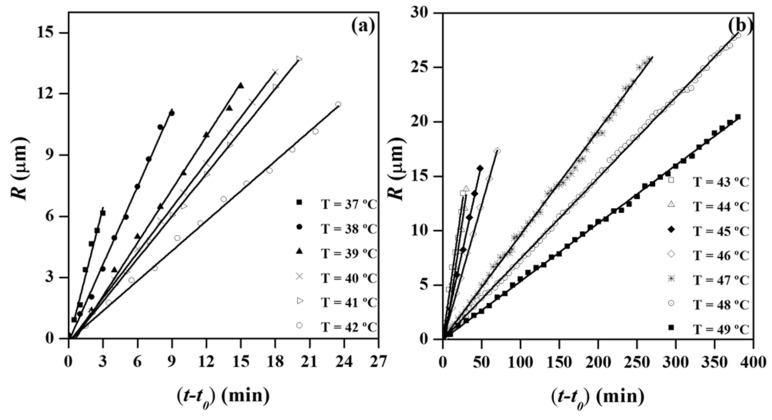
Variation of the spherulitic radius with crystallization time for temperatures between 37 °C and 42 °C (**a**) and 43 °C and 49 °C (**b**).

The radii of the spherulites grew linearly with time until impingement, as shown in Figure 13a,b for crystallization experiments performed in the above indicated temperature range. Final radii varied in this case between 6.5 and 27 μm and logically decreased at higher primary nucleation densities (i.e., at lower crystallization temperatures). Nevertheless, the spherulitic diameter was relatively small even at the higher assayed temperature since the nucleation density was still high.

It is worthy to note that a discontinuous growth of spherulites was not clearly detected and consequently a ridge and valley structure of the spherulite linked to depletion zones and fluctuations on the melt thin film, as postulated for P3HB polymers [33], could not be demonstrated in our case. Nevertheless, plots corresponding to higher temperature crystallized samples (e.g., 47 °C) showed small periodic fluctuations that although not being conclusive pointed towards the indicated interpretation. Small band spacing of P4HB and limitations of the experimental technique must be taken into account in this regard. 

### 3.7. Infrared Microspectroscopic Studies of P4HB Ringed Spherulites

**Figure 14:**
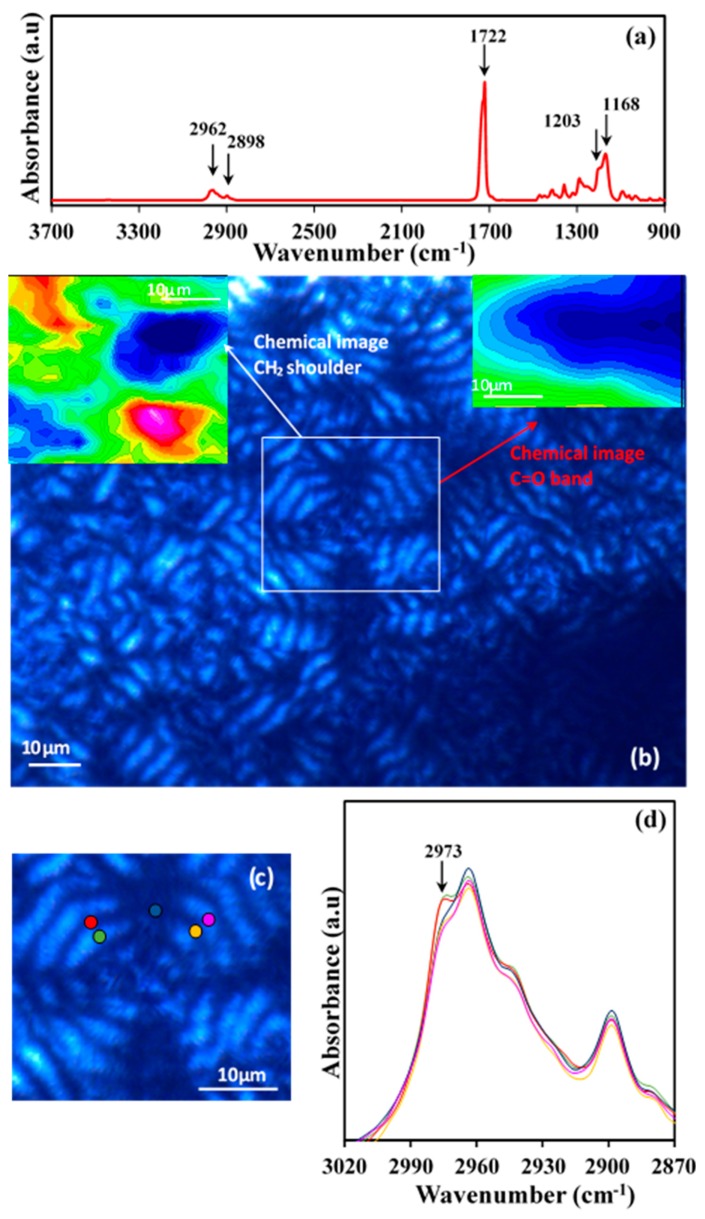
(**a**) FTIR spectra of THE P4HB suture with labelling of main bands; (**b**) Optical polarized micrographs showing spherulites developed in a solvent casting film. Insets show the chemical image obtained from C=O and CH_2_ (shoulder) bands; (**c**) Micrographs showing a representative banded spherulite and the specific microdomains were FTIR spectra were recorded; (**d**) Microinfrared spectra taken from the indicated microdomains (keeping the colour code). Different magnifications and wavenumber regions are showed.

Microspectroscopic experiments were undertaken in order to get insight on the hypothesis concerning the structure of ringed P4HB spherulites. Figure 14a shows the typical Fourier-transform infrared spectroscopy (FTIR) spectra of a commercial P4HB suture where characteristic stretching bands of methylene groups (2962 and 2898 cm^−1^) and ester groups (C=O at 1722 cm^−1^, and asymmetric and symmetric C–O at 1203 cm^−1^ and 1168 cm^−1^, respectively) can be detected.

Solvent casting of chloroform diluted solutions gave rise to films with a thickness lower than 5 μm that could be analyzed by transmission Fourier transform infrared microspectroscopy. Polarized optical microscopy images showed the development of spherulites (Figure 14b) with well differentiated dark and bright rings. Chemical images of the spherulites were obtained from the integration of the different infrared peaks (see insets of Figure 14b). In general, spherulitic sectorization was not observed in the chemical images when typical bands were considered, as for example the C=O band (i.e., see the inset of Figure 14b). In this case, the intensity of the peak was practically constant, with the only detected variation being a consequence of the not completely uniform thickness of the cast film. However, a detailed analysis was successful and a clear sectorization was derived by considering some minor intensity peaks as the high wavenumber shoulder of the 2973 cm^−1^ CH_2_ band. Interpretation of the peak is in progress, but the result points out the different orientation of the chemical bond that gives rise to a typical cross with two sectors with higher intensity (red) and two with the lower intensity (blue).

The indicated peak was also considered to discern, if some differences can be detected, between dark and bright rings. Figure 14c shows the specific microzones (colour points) that were analyzed and the corresponding microinfrared spectra. Main peaks had always the same intensity in agreement with the previous discussion and only small differences could be detected for the indicated minor peaks. These differences were only found along each specific ring (see shoulders corresponding to the zones indicated by the red, light blue and pink circles) but not along a specific spherulite radius. Thus, the indicated shoulder had the same intensity for dark (e.g., orange circle) and bright (e.g., violet circle) ring of a radius of the low intensity sector as well as for the dark (e.g., green circle) and bright (e.g., red circle) ring of a radius of the high intensity sector. In summary, the microinfrared spectra analysis is not consistent with a typical twisting of constitutive lamellae since clear differences should be expected between spectra recorded at determined radius as consequence of a periodic change on the orientation of chemical bonds. 

### 3.8. Secondary Nucleation Constant from DSC and Optical Microscopy Observations

The crystal growth rate, *G*, was analyzed by means of the Lauritzen-Hoffman (LH) equation in order to deduce the corresponding kinetic parameters [34]:

*G* = *G*_0_ × exp[−U*/(*R*(*T_c_* − *T*_∞_))] × exp[−*K_g_*/(*T_c_*(Δ*T*)*f*)] (7)

A similar equation can also be derived by considering the overall crystallization rate as deduced from DSC data:

*k* = *k*_0_ × exp[−U*/(*R*(*T_c_* − *T*_∞_))] × exp[−*K_g_*/(*T_c_*(Δ*T*)*f*)] (8)

The validity of this last approach implies a proportionality between *k* and *G* values, which in general is accepted for athermal crystallizations and obviously becomes progressively wrong as primary nucleation varied during the crystallization process [35,36,37,38].

**Figure 15:**
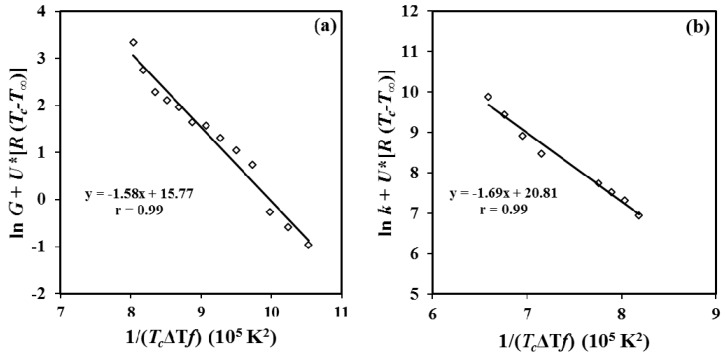
Lauritzen-Hoffman plots for the crystal growth rate (**a**) and the overall crystallization rate (**b**) for the isothermal crystallization of P4HB.

Following LH equation, the experimental spherulitic growth rates and overall growth rates of P4HB were plotted as ln *G* + U*/R(*T_c_* − *T*_∞_) versus 1/(*T_c_*(Δ*T*)*f*) (Figure 15a) and ln *k* + U*/*R*(*T_c_* − *T*_∞_) versus 1/(*T_c_*(Δ*T*)*f*) (Figure 15b). The plots were fitted with straight lines (*r^2^* = 0.99) when the “universal” values reported by Suzuki and Kovacs [39] (i.e., U* = 1500 cal/mol and *T*_∞_ = *T_g_* − 30 K) were used in the calculation. Kinetic features at low supercoolings are basically governed by the nucleation term, and consequently crystallization rates could become relatively insensitive to both U* and *T*_∞_ parameters. Plot allowed estimating secondary nucleation constants of 1.58 × 10^5^ K^2^ (from polarizing optical microscopy (POM) measurements) and 1.69 × 10^5^ K^2^ (from DSC data). The good agreement that is found allows to infer that the applied approximation (i.e., proportionality between *G* and *k*) seems correct and consequently the impact caused by the thermal nucleation is scarce. Probably, all primary nuclei became active when the exothermic crystallization peak starts to appear (i.e., after the induction time) and consequently a minimum discrepancy between the derived *K_g_* values is found.

**Figure 16:**
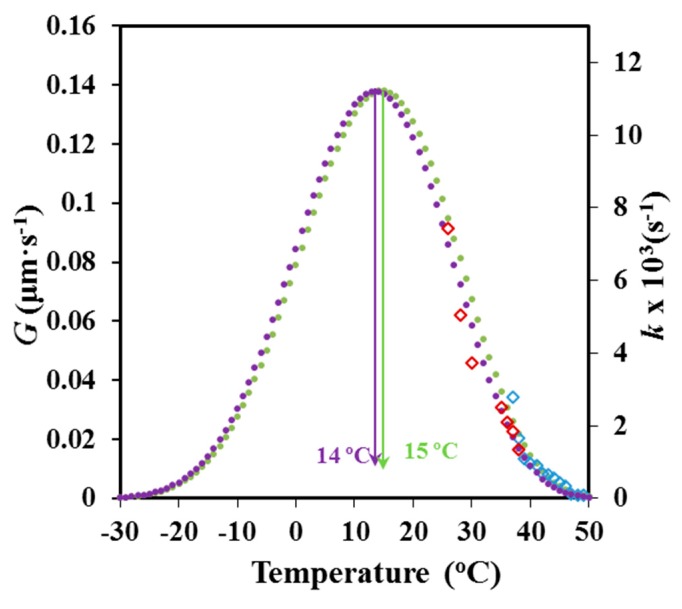
Temperature dependence of the crystal growth (●, **◊**) and overall crystallization (●, **◊**) rate. Simulated data is represented by circles and experimental data by rhombus.

Equation (7) and (8), and the estimated U*, *T*_∞_, *K_g_*, *G*_0_ and *k*_0_ parameters were used to estimate *G* and *k* values at different crystallization temperatures. The plot in Figure 16 shows, in both cases, the conventional bell-shapped curve expected from the interplay between segmental mobility and secondary nucleation. A satisfactory agreement was observed between the simulated curves obtained from DSC and POM data, as well as, between the limited experimental data and the simulated curves. The simulated curves also allowed estimating the crystallization temperatures at which crystal growth and overall crystallization rates became maxima (i.e., 14 °C (POM) and 15 °C (DSC). 

## 4. Conclusions

P4HB shows a complex melting peak associated to different lamellar populations and that is strongly dependent on the processing conditions. Thus, the temperature associated to the predominant melting peak can be increased by more than 12 °C after an annealing process. SAXS experiments demonstrated clear differences on the lamellar supramolecular order between annealed and melt crystallized samples. Lamellar thickness and contrast, between amorphous and crystalline regions, become higher for the annealed samples. These samples showed also a higher macroscopic crystallinity, although the order within lamellar stacks decreases due to the high increase of the amorphous lamellar thickness. An equilibrium melting temperature of 79.9 °C was deduced from the typical Hoffman-Weeks analysis.

DSC analysis of the isothermal crystallization of P4HB from the melt state allowed determining an average Avrami exponent of 2.56 and a single secondary nucleation constant of 1.69 × 10^5^ K^2^. P4HB experimented a cold crystallization process from the glassy state that occurred under high geometrical constrictions and lead to a decrease of the exponent to a value of 1.93.

Optical microscopy observations indicate that P4HB crystallized from the melt, gives rise to banded spherulites with clear differences between the widths of black and white rings and an interring spacing. Infrared microspectroscopy analysis indicated that chemical bonds had the same orientation in the dark and bright rings supporting a model based on a rhythmic growth. Primary nucleation increased by lowering the crystallization temperature, and even during an isothermal experiment. Nevertheless, the impact of the sporadic nucleation was reduced as could be deduced from the Avrami exponent and also by the value of the secondary nucleation constant (1.58 × 10^5^ K^2^) that is in full agreement with that determined from DSC experiments. Similar bell-shaped curves defined the dependence of the crystal growth and the overall crystallization rate, with 14–15 °C being the inferred temperatures for those associated to the maximum rates.

## Figures and Tables

**Table 1:** Isothermal crystallization kinetic parameters deduced from calorimetric (DSC) experiments for poly(4-hydroxybutyrate (P4HB).

*T_c_* (°C)	*n*	Z × 10^6^ (s^−*n*^)	*k* × 10^3^ (s^−1^)	*τ_1/2_* (s)
−26	1.95	33.50	5.13	164
−25	1.97	40.18	5.94	130
−24	1.91	70.68	6.71	113
−23	1.94	99.20	8.58	95
−22	1.93	134.25	9.89	78
−21	1.95	167.69	11.61	71
−20	1.84	381.86	13.98	65
28	2.35	3.99	5.03	177
30	2.52	0.74	3.72	392
35	2.71	0.09	2.49	341
36	2.63	0.09	2.10	382
37	2.56	0.10	1.84	481
38	2.62	0.03	1.35	722
